# Single-cell immune repertoire sequencing of B and T cells in murine models of infection and autoimmunity

**DOI:** 10.1038/s41435-022-00180-w

**Published:** 2022-08-26

**Authors:** Danielle Shlesinger, Kai-Lin Hong, Ghazal Shammas, Nicolas Page, Ioana Sandu, Andreas Agrafiotis, Victor Kreiner, Nicolas Fonta, Ilena Vincenti, Ingrid Wagner, Margot Piccinno, Alexandre Mariotte, Bogna Klimek, Raphael Dizerens, Marcos Manero-Carranza, Raphael Kuhn, Roy Ehling, Lester Frei, Keywan Khodaverdi, Camilla Panetti, Nicole Joller, Annette Oxenius, Doron Merkler, Sai T. Reddy, Alexander Yermanos

**Affiliations:** 1 Department of Biosystems Science and Engineering, ETH Zurich, Basel, Switzerland; 2 Department of Pathology and Immunology, University of Geneva, Geneva, Switzerland; 3 Institute of Microbiology, ETH Zurich, Zurich, Switzerland; 4 Department of Quantitative Biomedicine, University of Zurich, Zurich, Switzerland; 5 Division of Clinical Pathology, Geneva University Hospital, Geneva, Switzerland

**Keywords:** Clonal selection, Autoimmunity, Infection

## Abstract

Adaptive immune repertoires are composed by the ensemble of B and T-cell receptors within an individual, reflecting both past and current immune responses. Recent advances in single-cell sequencing enable recovery of the complete adaptive immune receptor sequences in addition to transcriptional information. Here, we recovered transcriptome and immune repertoire information for polyclonal T follicular helper cells following lymphocytic choriomeningitis virus (LCMV) infection, CD8+ T cells with binding specificity restricted to two distinct LCMV peptides, and B and T cells isolated from the nervous system in the context of experimental autoimmune encephalomyelitis. We could relate clonal expansion, germline gene usage, and clonal convergence to cell phenotypes spanning activation, memory, naive, antibody secretion, T-cell inflation, and regulation. Together, this dataset provides a resource for immunologists that can be integrated with future single-cell immune repertoire and transcriptome sequencing datasets.

## Introduction

B and T cells play a central role in orchestrating the immune response by recognizing foreign antigens through their B cell receptor (BCR; secreted version: antibodies) and T cell receptor (TCR), respectively. Upon primary antigen exposure, B and T cells rapidly undergo clonal expansion and can adopt a diverse set of cellular phenotypes with corresponding effector functions [1–3]. B cells further engage in germinal center (GC) reactions, where they undergo diversification through class-switch recombination and somatic hypermutation, hence increasing the range of effector functions and molecular recognition [4, 5]. B cells expressing high-affinity BCRs are selected for by T follicular helper (Tfh) cells, which are characterized by their expression of CD4, PD-1, CXCR5, and BCL6, and provide the required selection signals for B cell differentiation [6–13]. Following the resolution of immune challenges, and thereby the diminishing presence of cognate antigens, B and T cells contract and preferentially adopt heterogenous memory phenotypes characterized by expression of certain gene signatures [14, 15]. While this selection and differentiation of lymphocytes is heavily regulated under healthy conditions, dysfunctional clonal selection can contribute to autoimmunity and host pathology [16, 17].

The development of standardized protocols, reagents and dedicated commercial platforms (e.g., 10x Genomics) has made it possible to perform single-cell immune repertoire sequencing that simultaneously recovers gene expression (whole transcriptome) and immune repertoire (full-length, paired chains of BCRs (heavy and light chains) and TCRs (alpha and beta chains)) information [18–27]. This now enables investigation of how parameters such as clonal expansion relate to gene expression phenotypes [28], thereby providing novel insights not accessible by bulk sequencing (e.g., BCR variable heavy chain, TCR variable beta chain) methodologies [15, 29, 30]. In recent years, a number of studies have employed single-cell immune repertoire sequencing to dissect the heterogeneity and differentiation of T and B cells in contexts such as infection, disease, and vaccination [18, 25, 31–33].

Practical considerations such as the necessary cost and time required to generate and analyze each single-cell sequencing sample has been a limitation to performing large-scale experiments (i.e., with many samples). These challenges, combined with current practices of academic publishing, may result in studies with low sample numbers and the loss of publicly available data. Such problems can arise from a variety of sources including practical difficulties, day-to-day variation, technical problems such as machine failure, or poor experimental design. Despite this, pilot and potentially suboptimal experiments have the potential to nevertheless provide immunological insight and be used as a comparison to future experiments. Nevertheless, considerations such as batch correction, compatibility of integrated data and sensible interpretations need to be considered upon future reuse of data.

Here, we combine single-cell sequencing of both T and B cells from murine models of infection and autoimmune disease, exploring both gene expression and immune repertoire profiles. This dataset includes Tfh cells following acute and chronic infections with the model virus lymphocytic choriomeningitis virus (LCMV), CD8+ T cells with antigen-restricted specificity arising from spleens 15 months post infection with LCMV or murine cytomegalovirus (MCMV), thereby revealing distinct phenotypes of memory and inflationary T cells. These virus-specific T cells could be further compared to CD8+ T cells arising from the brain in a murine model of T-cell-driven neurological disease [34], which revealed gene expression patterns resembling T-cell exhaustion. Finally, we uncovered minimal transcriptional differences in a pilot study investigating the differences in BCR and TCR repertoires following induction of experimental autoimmune encephalomyelitis (EAE) using either myelin-oligodendrocyte glycoprotein (MOG) peptide (MOG35-55) or recombinant protein (rMOG). Our collection of single-cell immune repertoire sequencing experiments not only supports previously published results but also uncovers novel principles of B and T-cell clonal selection. Therefore, our dataset can serve as a reference for experimental and computational immunologists alike to inform future studies.

## Results

### Polyclonal Tfh cell populations following acute and chronic LCMV infection are transcriptionally heterogeneous, highly-expanded, and share clones with GP66-specific CD4+ T cells

**Fig. 1:**
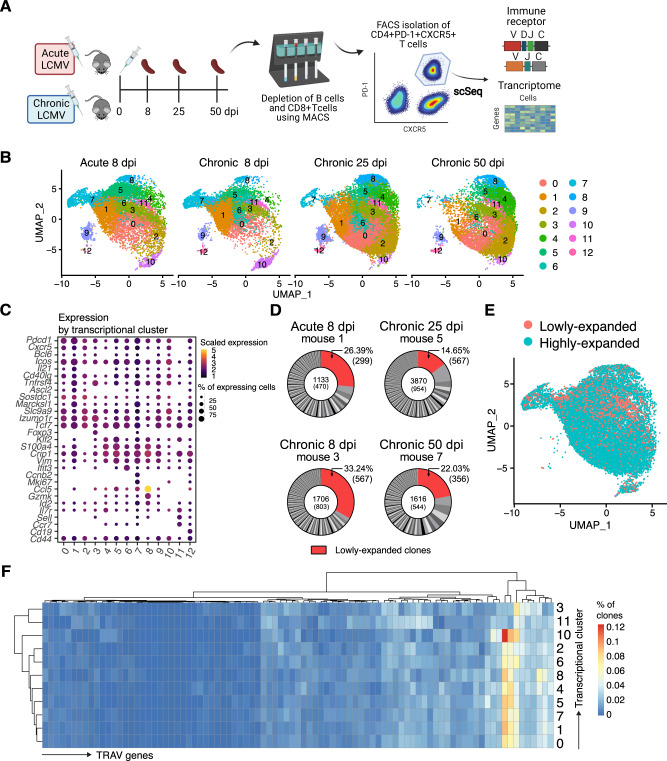
Different transcriptional phenotypes emerge in polyclonal Tfh cell populations following acute and chronic LCMV infection. **A** Experimental setup. CD4+CXCR5+PD-1+ cells were isolated from spleens of C57BL/6 mice infected with acute LCMV (*n* = 2) or chronic LCMV infection (*n* = 6). Each time point included two biological replicates. Cells were then sorted and sequenced to obtain both transcriptomes and TCR repertoires. **B** Uniform manifold approximation projection (UMAP) separated by experimental group. **C** Dottile plot depicting scaled expression of genes of interest separated by transcriptional clusters. **D** Representative donut plots depicting distribution of clonal expansion in each infection time point. Each section corresponds to a unique clone (defined by CDR3α-CDR3β nucleotide (nt) sequence) and the size corresponds to the fraction of cells relative to the total repertoire. Lowly-expanded clones (supported by only one unique cell) are colored in red. **E** UMAP displaying clonal expansion of Tfh cells after acute and chronic infection. Clones are defined by CDR3α-CDR3β nt sequence. Lowly-expanded clones are represented by only one unique cell, while highly-expanded clones are supported by more than one cell. **F** Heatmap depicting percentage of clones (defined by CDR3α-CDR3β nt sequence) using a particular TRAV gene (columns) in each cluster (rows).

Following immune challenges, B cells engage in GC reactions, where they undergo diversification through somatic hypermutation (SHM). B cells expressing high-affinity BCRs are selected for by Tfh cells and are characterized by their expression of CD4, PD-1, CXCR5, and BCL6 [6–10]. In an acute LCMV infection, viral clearance occurs within 8–10 days and is dependent on activation of cytotoxic CD8+ T cells [35, 36]. However, during a chronic LCMV infection, T-cell exhaustion occurs, making clearance dependent on late-emerging neutralizing antibodies that appear months after infection [37–39]. Tfh cells have been shown to be key players in the production of these late-emerging neutralizing antibodies and the resolution of chronic infection [40, 41]. To explore transcriptional and repertoire heterogeneity of Tfh cells during acute and chronic LCMV infection, we isolated Tfh cells at various time points post infection. We first depleted CD8+ T cells and B cells using magnetic-activated cell sorting (MACS) and subsequently used fluorescent activated cell sorting (FACS) to isolate CD4+CXCR5+PD-1+ cells from the spleens of C57BL/6 mice infected with LCMV causing acute (200 ffu LCMV clone 13 i.v., 8 dpi *n* = 2) or chronic disease (2 × 10^6^ ffu LCMV clone 13 i.v., 8, 25 and 50 dpi, *n* = 2 at each time point) [42]. We subsequently performed single-cell sequencing of the TCR repertoire and transcriptome using 10x genomics (Fig. 1A), which, following alignment to reference murine transcriptome, recovered gene expression data (GEX) for 55,440 cells with an average of 1186 median genes per cell (Fig. S1A).

We next used uniform manifold approximation projection (UMAP) to group cells with similar gene expression profiles into clusters (Fig. 1B), thus revealing a variety of transcriptional phenotypes such as “Tfh-like” (clusters 0–2; *Sostdc1*, *Marcksl1, Slc9a9*), Tfh and regulatory cells (cluster 3; Tfr; *Foxp3, Bcl6*), Th1- or T central memory precursor (Tcmp)like (clusters 4–5; *Crip1*, *Klf2*, *S100a4, Vim*), interferon-stimulated gene expressing (cluster 6; *Ifit3, Isg15*), proliferative (cluster 7; *Ccnb3, Mki67*), Th1-like (cluster 8; *Ccl5*, *Nkg7*, *Id2, Gzmk*), PI3K pathway expressing (cluster 10) and naive-like (cluster 11; *Sell*, *Ccr7, Il7r*, and low *Cd44*) (Figs. 1C and S1B–D). We additionally detected two clusters expressing genes associated with B cells and the complement cascade, suggesting minor contamination (clusters 9 and 12) (Fig. S1B). Cells belonging to mice sacrificed at 8 dpi with either acute or chronic LCMV (early time points of infection) occupied a distinct transcriptional space compared to cells belonging to mice with chronic LCMV sacrificed at 25 or 50 dpi (later time points of infection) (Fig. S2A). This was characterized by increased expression of genes associated with fatty acid metabolism, hypoxia and *Myc* targets, suggesting a more activated and proliferative phenotype early after infection (Fig. S2B). This is consistent with previous reports of Tfh cells proliferating 2 weeks after protein immunization [43].

We next aimed to explore the clonal diversity of Tfh cells during acute or chronic LCMV infection. For this, only cells expressing a single TCR alpha (α) and beta (β) chain were retained in the analysis and cells belonging to those clusters likely arising from contamination (9 and 12) were similarly excluded. We observed a polyclonal population of Tfh cells and an overall high degree of expansion in both acute and chronic LCMV infection (Figs. 1D and S2C). Notably, expansion was highest at 25 dpi with chronic LCMV and decreased again at 50 dpi, potentially representing T-cell contraction occurring as the infection is resolved [42]. We further analyzed the transcriptional space occupied by lowly- and highly-expanded clones (represented by one unique cell or more than one cell respectively), which revealed that Tfr cells and naive cells especially were lowly-expanded, although all clusters contained some lowly-expanded cells (Figs. 1E and S2D).

The abundance of certain TCR V germline genes or their pairings have been previously observed in virus-specific T cells in several infection conditions [15, 21, 29, 44–46]. Additionally, a previous study reported preferential usage of TRAV-TRAJ gene combination in Th1 and Tfh cells, indicating a contribution of the TCR α chain to lineage commitment [21]. This is congruent with previous findings demonstrating that Tregs have been reported to have distinct germline gene usage (e.g., dominated by TRBV5) and that Tfr have different TCR specificities than Tfh [47, 48]. Here, clusters 3 (Tfr) and 11 (naive-like) could be distinguished from other clusters by hierarchical clustering according to their TRAV gene usage (Fig. 1F), suggesting a difference in T-cell lineages compared to the other clusters. On an individual-chain level, similar TCR β V genes were expressed across all infection conditions (Fig. S3A, B), although on a clonal level, no group-specific pattern of TRAV-TRBV pairings could be observed (Fig. S3C). This is in contrast to previously reported results where TCR repertoires against a single peptide demonstrated stereotypic germline gene usage [46], suggesting that polyclonal repertoires encoding diverse TCR specificities are less dominated by certain combinations of germline genes.

**Fig. 2:**
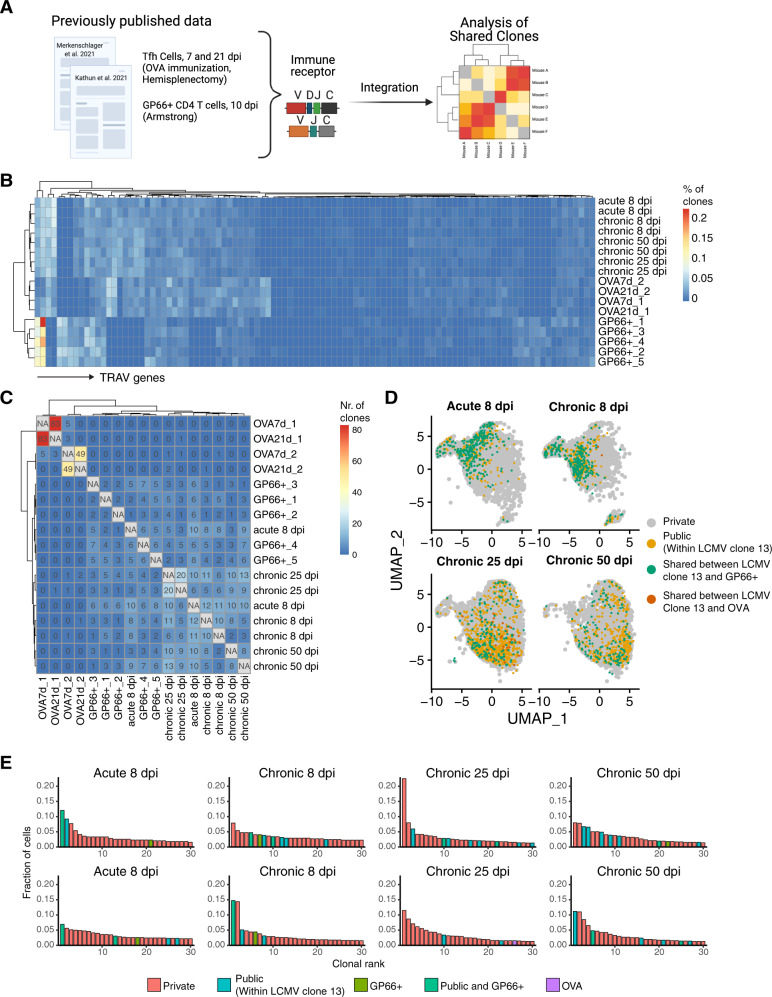
Polyclonal Tfh cells following acute and chronic infection include highly-expanded, public GPC-specific clones. **A** Graphical illustration of the integration of previously published data in order to investigate public clones: GP66-specific CD4+ cells from mice infected with Armstrong and sacrificed 8 dpi [21] and Tfh cells from OVA-immunized, hemi-splenectomized, mice (7 and 21 days post immunization) [43]. **B** Heatmap depicting percentage of clones (defined by CDR3α-CDR3β nt sequence) using a particular TRAV gene (columns) in each sample (rows). **C** Number of identical clones (defined by CDR3α-CDR3β aa sequence) found between Tfh cells from mice infected with LCMV clone 13, mice infected with Armstrong sacrificed 8 dpi [21] and Tfh cells from OVA-immunized, hemi-splenectomized, mice (7 and 21 days post immunization) [43]. **D** Uniform manifold approximation projection (UMAP) displaying shared clones between Tfh cells from mice infected with LCMV clone 13, mice infected with Armstrong sacrificed 8 dpi [21] and Tfh cells from OVA-immunized, hemi-splenectomized, mice (7 and 21 days post immunization) [43]. **E** Top 30 most expanded clones (defined by CDR3α-CDR3β aa sequence). Private clones are colored in red. Clones shared between mice infected with clone 13 (acute or chronic) are colored in blue and termed “Public”. Clones colored in green have inferred GP66 specificity. Clones colored in teal are “Public” and have inferred GP66 specificity. Purple colored clones are shared with OVA-immunized, hemi-splenectomized, mice (7 and 21 days post immunization) [43].

To investigate clonal convergence, we expanded our analysis by including previously published TCR repertoires of protein immunized mice and mice infected with a different LCMV strain. To this end, we integrated CD4+CD44+GP66+ T cells from mice infected with acute LCMV (Armstrong strain, 10 dpi) [21] and CD4+CD62^low^CD44^high^CXCR5^high^PD-1^high^BCL6+ Tfh cells from hemi-splenectomized mice following immunization with ovalbumin (OVA) (7 and 21 days post immunization) [43] (Fig. 2A). Hierarchical clustering according to TRAV gene usage separated the different datasets (Fig. 2B). Notably, Tfh cells from both virus infection and OVA immunization clustered in a separate node than CD4+CD44+GP66+ T cells, again in line with previous reports relating the TCR α chain to CD4+ cell fate decisions [21]. We then further explored repertoire similarities across mice in the context of shared clones after defining clones by identical CDR3α and CDR3β amino acid (aa) sequences. Mice infected with LCMV clone 13 (acute and chronic) shared up to 20 clones with each other and at least one clone from the GP66+ repertoires. Surprisingly, mice with chronic LCMV 8 and 25 dpi shared 6 clones with OVA-immunized mice (Fig. 2C). Visualizing all public clones on the UMAP showed that public clones were mainly absent from the Tfr and naive-like clusters (clusters 3 and 11) (Figs. 2D and S3D). As Tfr cells have been suggested to have autoreactive TCR specificities [47], our data suggest that Tfr clones might therefore also show a more personalized response.

We next questioned whether these repertoire similarities across mice were also observed in the context of public clones. We further expanded our analysis of clonal convergence by including previously published TCR repertoires of protein immunized mice or mice infected with a different LCMV strain. Next, we investigated the extent of clonal expansion of all shared clones by looking for public clones within the top 30 most expanded clones. Taking advantage of the known epitope specificity of GP66+ T-cell repertoires, we specifically highlighted clones shared with the GP66+ T-cell repertoires to infer TCR specificities. Several highly-expanded clones were public across all mice infected with LCMV clone 13 (Fig. 2E). Although we did not sort for virus-specific cells, the expansion of some virus-specific Tfh cells could nevertheless be confirmed, as several highly-expanded clones shared an identical CDR3α-CDR3β aa sequence with GP66+ T cells. Interestingly, one clone from the OVA-immunized mice was present in the top 30 most expanded clones of one repertoire arising 25 dpi with chronic LCMV (Fig. 2E). This particular clone, however, was represented by a single-cell in one of the OVA-immunized mice, which may arise due to the previously described bystander-activation that has been suggested to lead to antigen-unspecific Tfh cells in OVA-immunized mice [49]. Moreover, clonally expanded plasma cells with dual specificity to OVA and LCMV have previously been observed in chronically infected mice [25]. Together, this raises the question whether this particular clone is indeed specific to both LCMV or OVA and further motivates the exploration of TCR specificities of Tfh cells arising following immune challenges.

### Memory, inflationary, and exhaustion signatures of virus-specific CD8+ T cells

**Fig. 3:**
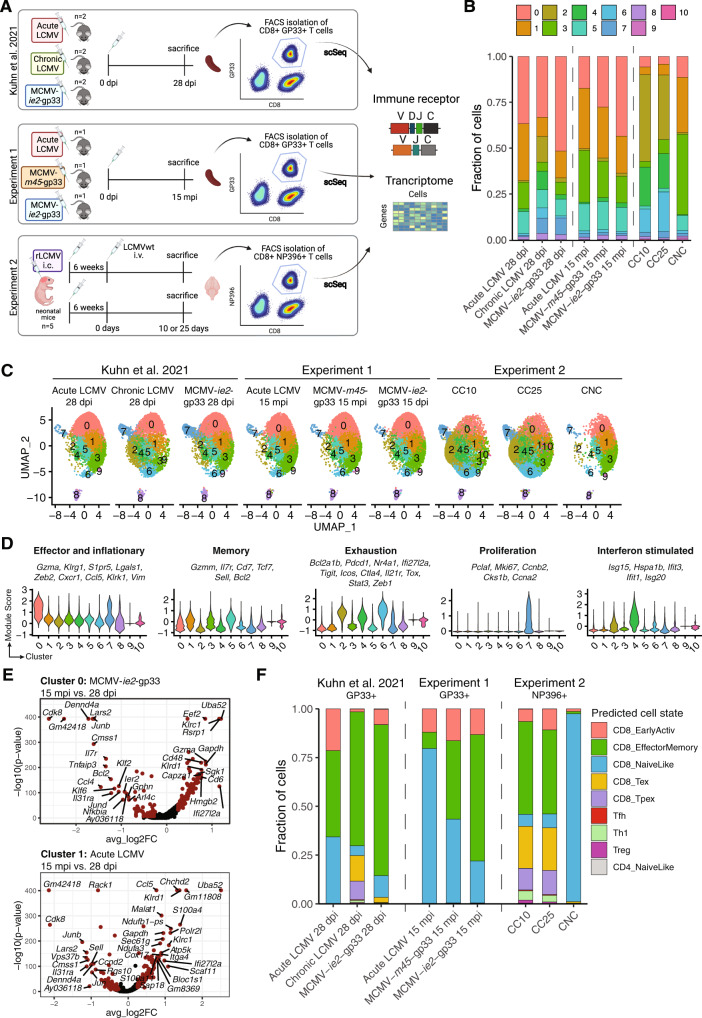
Virus-specific CD8+ T cells exhibit memory, inflationary, and exhausted phenotypes. **A** Experimental setup of each of the combined experiments. Previously published GP33-specific CD8+ cells from acute (*n* = 2) and chronic (*n* = 2) LCMV clone 13 and MCMV-*ie2*-gp33 (*n* = 2) infected mice 28 days post infection (dpi) were integrated with GP33-specific CD8+ cells from acute LCMV clone 13 (*n* = 1), MCMV-*m45*-gp33 (*n* = 1), and MCMV-*ie2*-gp33 (*n* = 1) infected aged mice (15 mpi). Additionally, NP396-specific CD8+ cells from carrier non-challenge (CNC) and carrier challenge 10 (*n* = 2) and 25 (*n* = 2) (CC10 and CC25, respectively) days post challenge from the viral déjà vu model. **B** Fraction of cells belonging to each cluster in each infection type. **C** Uniform manifold approximation projection (UMAP) split by infection type. **D** Module score for each cluster of genes associated with effector cell, memory, exhaustion, proliferative and interferon-expressing phenotypes. **E** Differential gene expression within cluster 0 between MCMV-*ie2*-gp33 infection 28 dpi and 15 mpi (top). Differential gene expression within cluster 1 between acute LCMV infection 28 dpi and 15 mpi (bottom). Points in red indicate differentially expressed genes (adjusted *p* value < 0.01 and average log2 fold change (FC) > 0.25). **F** Fraction of cells belonging to predicted cell state using the nearest-neighbor classifier of the ProjecTILs algorithm in each infection type.

We recently demonstrated that gp_33-41_-specific (GP33) CD8+ T cells adopted distinct transcriptional and selection phenotypes 28 days post acute, chronic, and latent viral infection [46]. We questioned whether infection-specific repertoire fingerprints remained stable 15 months post infection (mpi). We therefore performed single-cell immune repertoire sequencing on splenic GP33+CD8+ T cells from age-matched mice infected with either LCMV Armstrong or two distinct MCMV containing the LCMV gp_33-41_ peptide in either the *m45* or *ie2* loci (MCMV-*m45*-gp33 and MCMV-*ie2*-gp33, respectively) (Fig. 3A). Although both MCMV strains induce latent viral infections characterized by reactivation events, the location of gp_33-41_ allows for the isolation of CD8+ GP33-specific T cells that can be characterized by inflationary (using MCMV-*ie2*-gp33) and non-inflationary (using MCMV-*m45*-gp33) T-cell phenotypes [15]. This provided an age-matched comparison of virus-specific CD8+ T cells either lacking antigen stimulation versus repeated exposure to their cognate antigen. Overall, we recovered GEX data for 24,381 cells with an average of 956 median genes per cell (Figs. 3A and S4A). Additionally, we compared these cell phenotypes to T cells arising from the viral déjà vu model, which has previously been leveraged to induce T-cell driven CNS inflammation [34, 50, 51]. In this model, neonatal intracranial (i.c.) infection of an attenuated strain of LCMV (rLCMV) causes CNS-restricted viral persistence which leads to the development of immune-driven CNS disease following an i.v. infection with wild type LCMV (wtLCMV). We performed single-cell immune repertoire sequencing on immunodominant NP396+CD8+ T cells of mice receiving only a neonatal i.c. infection (carrier-non-challenge (CNC), *n* = 1) and mice receiving both an i.c. infection and an i.v. infection with wtLCMV following 6 weeks and sacrificed after either 10 or 25 days (Carrier challenge [CC10 and CC25, respectively], *n* = 3 for each time point) (Fig. 3A). Here, we recovered GEX data for 36,590 cells with an average of 1001 median genes per cell (Fig. S4A). We then performed unsupervised clustering and UMAP on cells from both experiments in addition to our recently published GP33-specific CD8+ T cells 28 dpi [46]. Since batch-effects between experiments could be observed when using feature-level scaling for integration (Fig. S4B), the harmony integration method was used for batch correction [52, 53]. This resulted in ten clusters, nine of which contained cells from all three experiments (Fig. 3B, C). We next investigated the cluster-defining genes in an unbiased manner, thus observing clusters enriched in genes associated with phenotypes such as effector and inflationary (*Klrg1, Gzma, Zeb2)*, memory (*Il7r, Sell, Bcl2*), exhaustion (*Pdcd1, Ctla4, Tigit, Stat3, Tox*), interferon-stimulated gene expressing (*Isg15, Ifit3*) and proliferation (*Pclaf, Mki67, Ccnb2*) (Figs. 3D and S4B). Even 15 mpi, cluster 0 expressing genes associated with the previously reported effector-memory phenotype of inflationary CD8+ T cells could be clearly observed [15] (Figs. 3B–D and S4B). Memory clusters 1 and 5 were represented by a higher proportion of cells at 15 mpi and CC25 compared to their earlier infection counterparts. Notably, chronic LCMV infection 28 dpi as well as CC10 and CC25 had a higher fraction of cells belonging to cluster 2 and 7, expressing genes associated with exhaustion and proliferation, respectively. Comparing age-matched mice within cluster 0 (effector and inflationary) and cluster 1 (memory), revealed *Cdk8*, *Lars2*, *Junb* and *Il31ra* being differentially expressed amongst others in LCMV and MCMV-*ie2* infected mice 15 mpi, while *Ccl5, Uba52 and Ifi27l2a* were differentially expressed in both infections 28 dpi. This points toward potentially age-associated gene signatures with 100 and 52 differentially expressed genes shared between same-aged mice and an enrichment of effector associated phenotype 28 dpi within both clusters (Figs. S4E and S5A). Next, we used the nearest-neighbor classifier of the ProjecTILs algorithm [54] to predict cell states within our data. ProjecTILs enables the projection of new scSeq data onto a previously generated and annotated reference T-cell atlas and can therefore classify common T-cell subsets [54]. High proportions of predicted CD8+ effector memory cells could be detected in all mice, with the exception of 15 mpi with acute LCMV and CNC (Figs. 3F and S5B). Although ProjecTILs predicted 2034 out of 31,681 cells to have CD4+ T cell phenotypes, this did not match the minor expression patterns of CD4+ T cells present in the data (Fig. S5C). The fraction of cells predicted to be CD8+ decreases at 15 mpi compared to their age-matched counterparts, again potentially representing the contraction occurring in virus-specific T cells [55]. Moreover, acute LCMV infections and CNC had high proportions of cells with a predicted CD8+ naive phenotype, which under experiment conditions most likely represents central memory cells [54]. Both carrier-challenged mice showed higher proportions of T cells with a predicted exhaustion or exhausted precursor phenotype, even exceeding those arising from chronically infected mice 28 dpi (Figs. 3F and S5B). Trajectory analysis for clone-specific differentiation pathways for Tfh cells and virus-specific CD8+ T cells following acute and MCMV-*ie2*-gp33 infection revealed phenotypic (effector versus memory) differences in early and late time points in terms of pseudotime location for both Tfh and GP33-specific CD8+ T cells following LCMV infection. No distinct trajectory between virus-specific T cells isolated at early and late time points following MCMV infection could be observed. Consistent with this, we could further observe fluctuating gene expression patterns across pseudotime for the most expanded Tfh and CD8+ T cells arising from LCMV infection (Fig. S6A). We were finally interested in comparing sequence motifs of repertoires with various epitope-specificities and polyclonal repertoires. Comparison of the complementarity determining region 3 (CDR3) of TCR α and TCR β from GP33- and NP396-specific cells, as well as the previously discussed polyclonal Tfh cells suggested biochemical differences between the groups (Fig. S7A), supporting a previous report that biochemical properties of TCR-interactions may dictate cell fate [21].

### Clonally expanded T cells persist into old age and retain memory and inflationary phenotypes following acute and latent viral infections

**Fig. 4:**
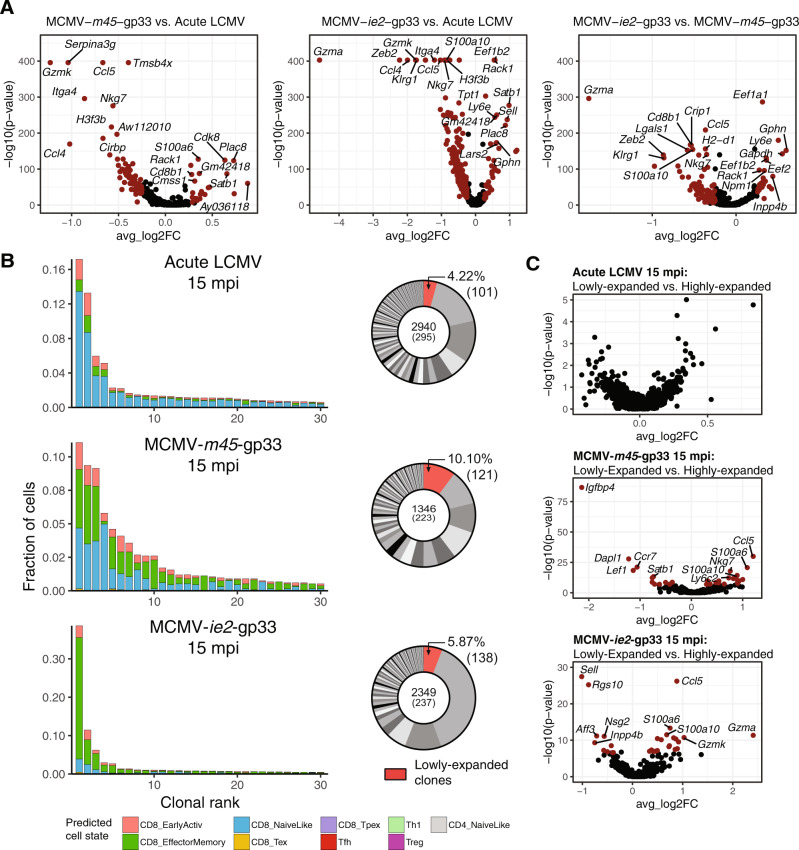
Memory and inflationary phenotypes in aged mice following acute and latent viral infections. **A** Differential gene expression between aged mice. Points in red indicate differentially expressed genes (adjusted *p* value <0.01 and average log2 fold change (FC) > 0.25). **B** Top 30 most expanded clones of aged mice separated by the predicted cell state using the nearest-neighbor classifier of the ProjecTILs algorithm [54]. Distribution of clonal expansion is represented by donut plots. Each section corresponds to a unique clone (defined by CDR3α-CDR3β nt sequence) and the size corresponds to the fraction of cells relative to the total repertoire. Lowly-expanded clones (supported by only one unique cell) are colored in red. **C** Differential gene expression within infection type between lowly- (=1 cell) and highly-expanded (>1 cell) clones.

After analyzing the global transcriptional landscape of our virus-specific CD+8 T-cell datasets, we questioned the extent of transcriptional heterogeneity within the different T-cell populations persisting 15 mpi. Performing differential gene expression analysis between the aged groups emphasized the presence of the inflationary T-cell phenotype (e.g., expression of *Klrg1, Gzma, Zeb2*) following infection with MCMV-*ie2-*gp33 compared to mice infected with acute LCMV or MCMV-*m45*-gp33. (Fig. 4A). Moreover, non-inflationary T cells differentially expressed genes associated with effector phenotypes (*Gzmk, Ccl4*) compared to acute LCMV infection (Fig. 4A). Having observed polyclonal but private clonal expansion in virus-specific cells 28 dpi, we questioned the extent to which clonally expanded cells were present in mice 15 mpi. Quantifying the fraction of the total repertoire composed by each clone demonstrated that highly-expanded clones represented the vast majority of the repertoire for all three mice (Fig. 4B). Interestingly, the most expanded clone in MCMV-*ie2*-gp33 infected mice represented ~40% of the total recovered repertoire (Fig. 4B). Analysis using ProjecTILs revealed a high proportion of CD8+ effector memory cells in MCMV-infected mice compared to acute LCMV infection. Notably, MCMV-*m45*-gp33 infected mice had increased proportions of predicted naive-like cells (potentially representing central-memory cells) within the most expanded clones, again differentiating between inflationary and non-inflationary T cells (Fig. 4B). Compared to 28 dpi infection, the most expanded clones 15 mpi showed increased proportions of predicted naive-like cells in the case of acute infection. However, in MCMV-*m45*-gp33 infection the most expanded clones were more phenotypically similar between time points, with relatively less clonal expansion present 15 mpi (Fig. S7B, C). Comparison of sequence motifs demonstrated less CDR3-encoded diversity at 15 mpi compared to 28 dpi (Fig. S7D). We have previously reported that highly-expanded clones have relatively higher expression of effector molecules, such as *Nkg7*, *Ccl5*, and granzymes [18, 19]. This was consistent in MCMV-infected mice, however, was not observed at 15 mpi with acute LCMV infection.

### Carrier-challenged NP396-specific CD8+ cells retain an exhausted phenotype 25 days post viral challenge

**Fig. 5:**
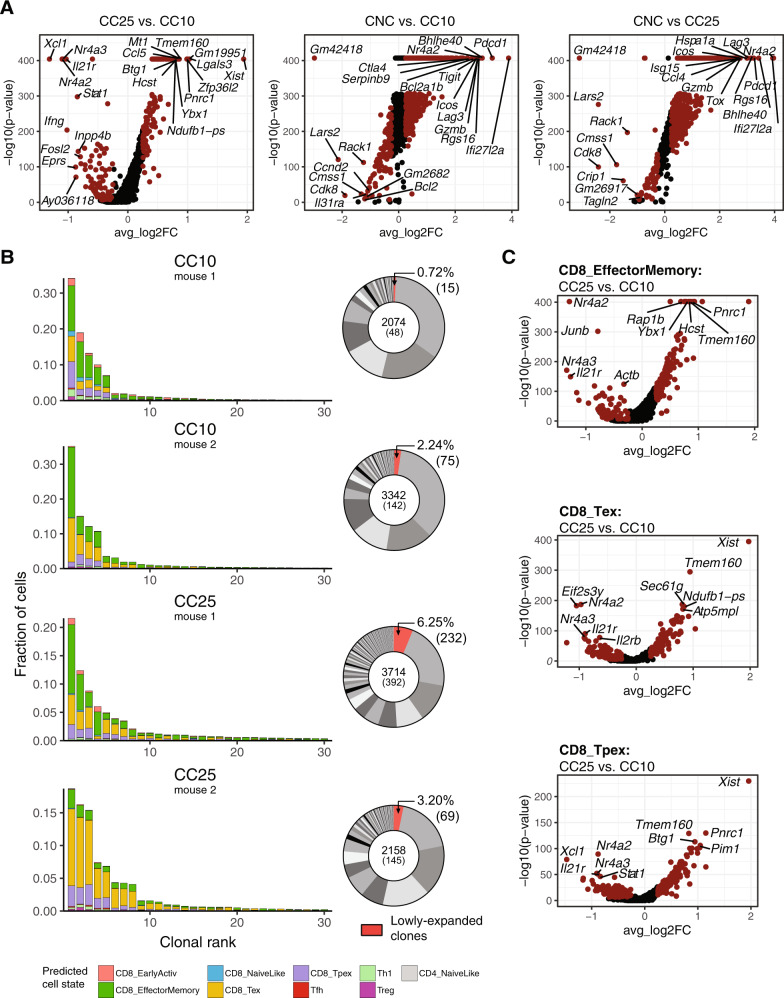
Carrier-challenged (CC) NP396-specific CD8+ exhibit exhausted cell signatures up to 25 days post viral challenge. **A** Differential gene expression between mice from the viral déjà vu model. Points in red indicate differentially expressed genes (adjusted *p* value < 0.01 and average log2 fold change (FC) > 0.25). **B** Top 30 most expanded clones of mice from the viral déjà vu model separated by the predicted cell state using the nearest-neighbor classifier of the ProjecTILs algorithm [54]. Distribution of clonal expansion is represented by donut plots. Each section corresponds to a unique clone (defined by CDR3α-CDR3β nt sequence) and the size corresponds to the fraction of cells relative to the total repertoire. Lowly-expanded clones (supported by only one unique cell) are colored in red. **C** Differential gene expression within predicted cell states between CC25 and CC10.

In the viral déjà vu model, it has been reported that interferon-γ (IFN-γ) expression by CD8+ T cells and the resulting neuronal STAT1/CCL2 signaling play a key role in disease development [34, 51]. However, the transcriptional programs and clonality of these virus-specific T cells have not been comprehensively profiled at a single-cell resolution. Performing differential gene expression analysis between all infection conditions revealed exhaustion and effector phenotypes (genes) in both CC10 and CC25 mice compared to CNC mice. Interestingly, both *Stat1* and *Ifng* were upregulated in CC25 compared to CC10 mice, despite earlier time points being associated with more severe clinical disease scores [34, 51]. To explore clonal expansion during carrier challenge, we quantified the fraction of the total repertoire composed by each clone. High levels of clonal expansion could be observed in all four mice. Within the top 30 most expanded clones, the majority of cells were predicted to have exhausted phenotypes by the ProjecTILs algorithm (Fig. 5B). Moreover, even within the exhausted and effector memory cells, genes associated with TCR signaling (*Nra4a2, Nra4a3, Il21r, Stat1)* were differentially expressed 25 days post viral challenge.

### Lymphocytes are transcriptionally similar following EAE induction with either rMOG and MOG33-55

**Fig. 6:**
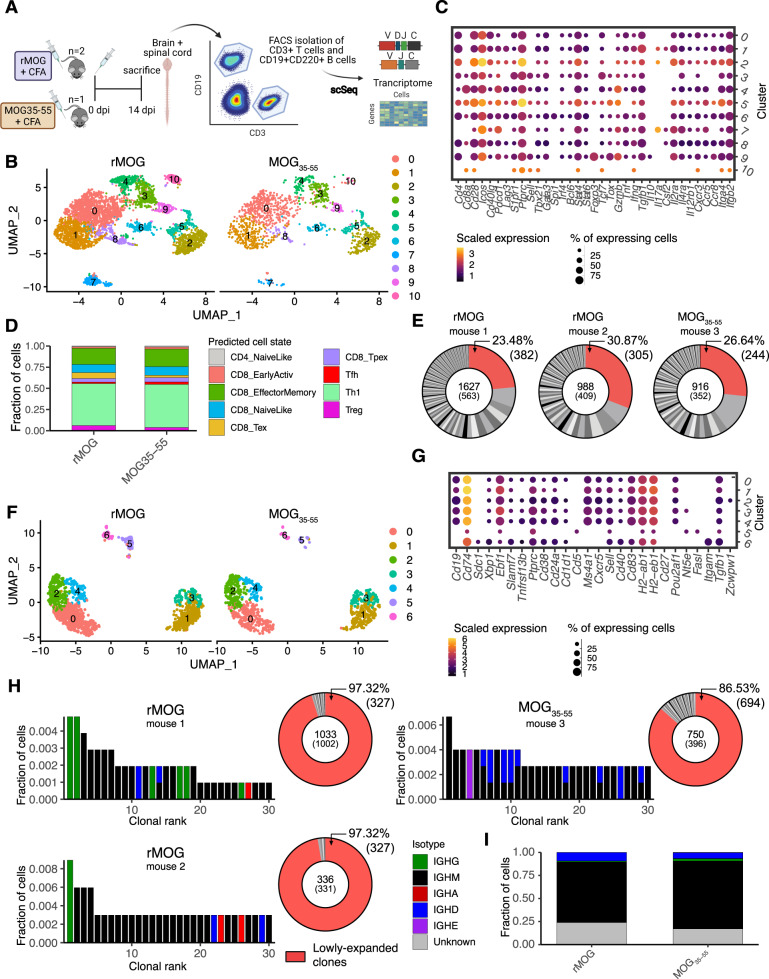
Transcriptional similarity of B and T cells following experimental autoimmune encephalomyelitis (EAE) induction with either rMOG and MOG33-55. **A** Experimental setup of single-cell immune repertoire sequencing. CD3+ T and CD19+CD220+ B cells were isolated from mice immunized with either recombinant myelin oligodendrocyte protein extracellular domain aa1-121 (rMOG, *n* = 2) or the peptide MOG35-55 (*n* = 1), after 14 days. **B** Uniform manifold approximation projection (UMAP) of T cells split by immunization type. **C** Dottile plot showing genes of particular interest in all clusters arising from unsupervised clustering in T cells. **D** Fraction of T cells belonging to predicted cell state using the nearest-neighbor classifier of the ProjecTILs algorithm [54] for both groups. **E** Donut plots representing clonal expansion of T cells. Each section corresponds to a unique clone (defined by CDR3α-CDR3β nt sequence) and the size corresponds to the fraction of cells relative to the total repertoire. Lowly-expanded clones (supported by only one unique cell) are colored in red. **F** Uniform manifold approximation projection (UMAP) of B cells split by experimental group. **G** Dottile plot showing genes of particular interest in all clusters arising from unsupervised clustering in B cells. **H** Top 30 most expanded B cell clones of mice immunized with either rMOG or MOG35-55 colored by isotype. Distribution of clonal expansion is represented by donut plots. Each section corresponds to a unique clone (defined by CDRH3-CDRL3 nt sequence) and the size corresponds to the fraction of cells relative to the total repertoire. Lowly-expanded clones (supported by only one unique cell) are colored in red. **I** Fraction of B cells corresponding to a specific isotype. Cells with either less or more than one heavy and one light chain were assigned an “unknown” isotype.

We finally investigated B and T cells during EAE, a mouse model for the autoimmune neuroinflammatory disease multiple sclerosis [17]. In this model the autoreactive immune response can be elicited by immunization with a myelin-derived antigen coupled with an adjuvant. To this end, the recombinant myelin oligodendrocyte protein extracellular domain aa1-121 (rMOG) or the peptide MOG35-55 can be used amongst others. Notably, it has been suggested that B cells play differing roles in CNS pathology depending on whether rMOG or MOG35-55 is used [56, 57]. Here, we performed single-cell sequencing of transcriptome and immune repertoires from CD3+ T and CD19+CD220+ B cells from the brains and spinal cords of mice immunized with rMOG + CFA and MOG35-55 + CFA, to compare how rMOG and MOG35-55 differentially imprint immune repertoires in the context of EAE (Fig. 6A). To this end, we recovered GEX data for 5444 T cells and 2047 B cells with an average of 1355 median genes per cell (Fig. S6A). We first investigated common T-cell markers and transcriptional cluster membership, which demonstrated similar expression patterns between rMOG and MOG35-55 groups (Figs. 6B, C and S6B). Comparing cell states predicted using the ProjecTILs algorithm [54], similarly revealed high congruence between peptide- and protein-induced EAE (Figs. 6D and S6C), with CD8+ effector memory and Th1 cells composing the majority of predicted cell states. Similar to the other T-cell repertoires described within this study, we observed high levels of clonal expansion (Fig. 6E). We further investigated whether gene signatures specific to expansion were present, which suggested that expanded CD4+ and CD8+ expanded T cells adopted a tissue-resident effector memory signature, associated with decreased expression of *Ilr7*, *Tcf7* and *S1pr1* and higher expression of *Cxcr6* (Fig. 6S). A similar transcriptional signature was previously observed in expanded T cells in human MS patients [58]. Expression of markers for mature B cells, such as *Cd19*, *Ebf1*, *Ms4a1* (encoding CD20) and *Ptprc*, was high in all clusters excluding clusters 5 and 6 (Fig. 6F). After having observed transcriptional similarity across samples, we questioned whether similar patterns were also present in B cells. Cells from both immunizations were present in all resulting transcriptional clusters (Fig. 6E). The clonal expansion of B cells expressing a single heavy and light chain was lower compared to T cells, while expansion between immunization strategies did not differ (Fig. 6H). Assessing the isotypes of the top 30 most expanded B cells clones, in addition to all clones, revealed high frequencies of the IgM isotype (Fig. 6G, H), which is similar to previous findings profiling B cells in the CNS of naive mice [18].

## Discussion

Single-cell sequencing of lymphocytes now makes it possible to profile adaptive immune repertoires and their transcriptomes at high-throughput. Immune repertoire features such as clonal expansion, germline gene usage, and transcriptional phenotypes provide a quantitative overview of an immune response and hold the potential to infer immunological status. Despite a growing number of studies leveraging single-cell immune repertoire sequencing, some pilot and small-scale sequencing experiments do not reach the public domain despite the potential to both uncover immunological principles and integrate with larger public datasets. Although low numbers of mice limit the conclusions that can be made by this study, our single-cell sequencing data presented can nevertheless be used in combination with other immune repertoire data as demonstrated by multiple of our conclusions aligning with previously published results. Examples of this include transcriptional differences between early and later time points of Tfh [59], GP66-specific TCRs detected in Tfh following LCMV infection [21], distinct repertoire features of Tfr [47], inflationary and exhaustion phenotypes of virus-specific CD8+ T cells [15, 50], and minor differences between B and T cells when inducing EAE with either rMOG or MOG35-55 [60]. In addition to these confirmatory findings, our collection of single-cell immune repertoire sequencing can spark future experiments investigating clonal selection of lymphocytes across various experimental models. These include relating antigen specificity to expansion and expression phenotypes of Tfh populations during chronic infection, quantifying repertoire dynamics relating to Tfh and Tfr during GC collapse, exploring consequences of prolonged T-cell exhaustion and effector phenotypes persisting beyond the peaks of neurological disease (déjà vu) and latent viral infections, or revisiting previously reported differences between MOG peptide and protein models of EAE in terms of the adaptive immune response [56].

## Methods

### Mice experiments

All animal experiments were performed in accordance with institutional guidelines and Swiss federal regulations. Experiments involving LCMV, MCMV-*ie2*-gp33, MCMV-*m45*-gp33 infections were approved by the veterinary office of the canton of Zurich or Geneva under animal experimentation licenses ZH114/2017, ZH115/2017 and GE170-19. Female C57BL6J WT mice between the age of 8–12 weeks were used for all experiments unless otherwise stated. Acute LCMV infections were infected intravenously (i.v.) with 200 focus forming units (ffu) of LCMV clone 13 in the tail vein. Latent infections were established by injecting 2 × 10^5^ pfu dose of MCMV-*ie2*-gp33 or MCMV-*m45*-gp33 i.v., which were obtained from Dr. L. Cicin-Sain and contained a functional *m157* gene as previously described [61]. MCMV viral stocks were propagated on M2-10B4 cells and purified by ultracentrifugation using a 15% sucrose gradient. LCMV clone 13 was produced as previously described [2, 39]. Upon sacrifice 28 dpi or 15 mpi, organs were harvested and single-cell suspensions were prepared by mashing the tissue through a 70 µM cell strainer and rinsing with complete RPMI (RPMI-1640 supplemented with 10% fetal bovine serum, 2 mM L-glutamine, 1% penicillin-streptomycin, 1 mM sodium pyruvate, 50 nM beta-mercapthoethanol, 0.1 mM non-essential [glycine, L-alanine, L-asparagine, L-aspartic acid, L-glutamic acid, L-proline, L-serine] amino acids, 20 mM HEPES). For the isolation of Tfh cells, MACS was performed according to manufacturer’s protocol (Miltenyi Biotec, MACS Cell Seperation) to remove CD8+ and B cells. The single-cell suspension was then stained with Live-Dead (APC-Cy7), CD4 (APC), PD1 (PE-Cy7) and CXCR5 (PE) and cells were isolated by flow cytometric sorting (FACSAria and FACSDiva software). In the case of antigen specific CD8+ T cells the single-cell suspension was then incubated with CD8-PE (clone 53-6.7, Biolegend), MHC class 1 tetramer for gp_33-41_ conjugated to APC diluted in FACS buffer (PBS, 2 mmEDTA, 2% FCS) at room temperature for 30 min, as previously described [62], and LiveDead nearIR (Thermo Fisher). Tetramer-positive cells were isolated via flow cytometric sorting (FACSAria with FACSDiva software) and subsequently supplied as input for single-cell immune repertoire sequencing. Recombinant LCMV strains and viral déjà vu infections were set up as previously described [34]. EAE was induced by the injection of either 325 μg rMOG or 200 μg MOG35-55 in CFA as previously described [63]. Following sacrifice, mice were transcardially perfused with 4 °C cold PBS for 3 min. Brains and spinal cords were collected in RPMI, transferred to a tube containing 1 ml RPMI with 1.0 mg/ml Collagenase I/DNAse I, cut into small pieces, incubated for a further 30 min at 37 °C and mashed through a 70 μm cell strainer. Single-cell suspensions of brains and spinal cords from each mouse were pooled in the case of rMOG. Lymphocytes from brain and spinal cord for déjà vu and EAE models were isolated using 30 and 70% Percoll gradients by centrifugation. Cells were stained with anti-CD3-FITC, anti-B220-APC and anti-CD19-PE-Cy7 and sorted on a FACSAria 3 (BD Biosciences) as B cells (CD19+, B220+) or T cells (CD3+, CD19−) into 1.5 ml tubes.

### Single-cell immune repertoire analysis

Single-cell immune repertoire sequencing was performed as according to the 10x Genomics Chromium Single-Cell V(D)J Reagents Kit (CG000166 Rev A) as previously described [25]. In brief, single cells for all samples were simultaneously encapsulated with gel emulsion microdroplets (10x Genomics, 1000006) in droplets using a Chromium Single-Cell A Chip (10x Genomics, 1000009) with a target loading of 13,000 cells per reaction. cDNA amplification was performed using 14 cycles and subsequently split for downstream GEX and VDJ library preparation. GEX libraries were amplified using the Chromium Single-Cell 5’ Library Kit (10x Genomics, 1000006). TCR libraries were amplified using the Chromium Single-Cell V(D)J Enrichment Kit, Mouse T-Cell (10x Genomics, 1000071). Final libraries were pooled and sequenced on the Illumina NovaSeq S1 using a concentration of 1.8 pM with 5% PhiX. Paired-end sequencing files for GEX and VDJ libraries were aligned to the murine reference genome (mm10) and V(D)J germlines (GRCm38) using 10x Genomics cellranger (v5.0.0) count and vdj arguments, respectively. The filtered feature matrix directory was supplied as input to the VDJ_GEX_matrix function in the R package Platypus (v3.2.1) [19], which uses the transcriptome analysis workflow of the R package Seurat [53]. Only those cells containing less than 20% of mitochondrial reads were retained in the analysis. Genes involved in the adaptive immune receptor (e.g., TRB, TRBV1-1), were removed from the count matrix to prevent clonal relationships from influencing transcriptional phenotypes. Gene expression was normalized using the “scale.data” (Tfh, EAE) or “harmony” (GP33+ aged, déjàvu) argument in the VDJ_GEX_matrix function. 2000 variable features were selected using the “vst” selection method and used as input to principal component analysis using the first ten dimensions. Graph-based clustering using the Louvain modularity optimization and hierarchical clustering was performed using the functions FindNeighbors and FindClusters in Seurat using the first ten dimensions and a cluster resolution of 0.5. UMAP was similarly inferred using the first ten dimensions. The FindMarkers function from Seurat was used when calculating differentially expressed genes (both across groups or across clusters) with logfc.threshold set to 0 and minimum number of cells expressing each gene set to 0.25 and subsequently supplied to the GEX_volcano and GEX_gsea functions from Platypus. Mitochondrial and ribosomal genes were removed when visualizing DE genes. Gene expression heatmaps, feature plots, and violin plots were produced by supplying genes of interest to the functions DoHeatmap, FeaturePlot, and VlnPlot functions in Seurat, respectively. Module score was calculated using the AddModuleScore from Seurat. The GEX_gsea function uses the R package fgsea (v1.16.0) (Sergushichev 2016), which performs gene set enrichment analysis (GSEA) and uses the adaptive multilevel splitting Monte Carlo approach. Hallmark (H) gene sets from the Molecular Signatures Database (MSigDB) were used for GSEA [64]. Cells containing no or more than one α/heavy and β/light chain were filtered out for TCR/BCR repertoire analysis. Clones were defined by identical CDR3α/CDRH3 and CDR3β/CDRL3 sequence (nucleotide or amino acid sequence) across all repertoires. Clones represented by more than one cell were considered highly-expanded clones, while single-celled clones were defined as lowly-expanded. The VDJ_circos function from Platypus was used to create circos plots. The R package pheatmap (v1.0.12) was used to create heatmaps displaying V gene usage or number of shared clones [65]. The projection of cells onto reference UMAPs and cell state predictions were done using the R package ProjecTils (v2.2.0) [54], Each experiment was projected individually onto the tumor-infiltrating T lymphocytes (TIL) atlas. Trajectory analysis was pefromed by transforming the separate Seurat Objects into a Cell Data Set using as.cell_data_set function from the SeuratWrappers package (v0.3.0) and subsequently supplying it as input to Monocle3 (v1.2.9) to calculate the trajectories [66–68].

## Supplementary information

Supplementary Figures

## Data Availability

All GEX and VDJ immune repertoire sequencing data have been deposited on the EMBL’s European Bioinformatics Institute website under the accession code E-MTAB-11330. Donut plots were created using GraphPad Prism® Software version 9. The packages ggplot (v3.3.3), ggrepel (v0.9.1), ggpubr (v0.4.0), cowplot (v1.1.1) and gridExtra (v2.3) were used for data visualization. Experimental overviews were created with BioRender.com.

## Code availability

Code and functions used in this study are available at https://github.com/alexyermanos/Platypus.
