# Supplementary material for: Single-cell immune repertoire sequencing of B and T cells in murine models of infection and autoimmunity
Source: Genes Immun. 2022 Aug 26;23(6):183–95. doi: 10.1038/s41435-022-00180-w (PMC9519453; doi:10.1038/s41435-022-00180-w)
Supplement: Supplementary file 1 — Supplementary Figures [file 41435_2022_180_MOESM1_ESM.docx]

**Supplementary Figures**

**
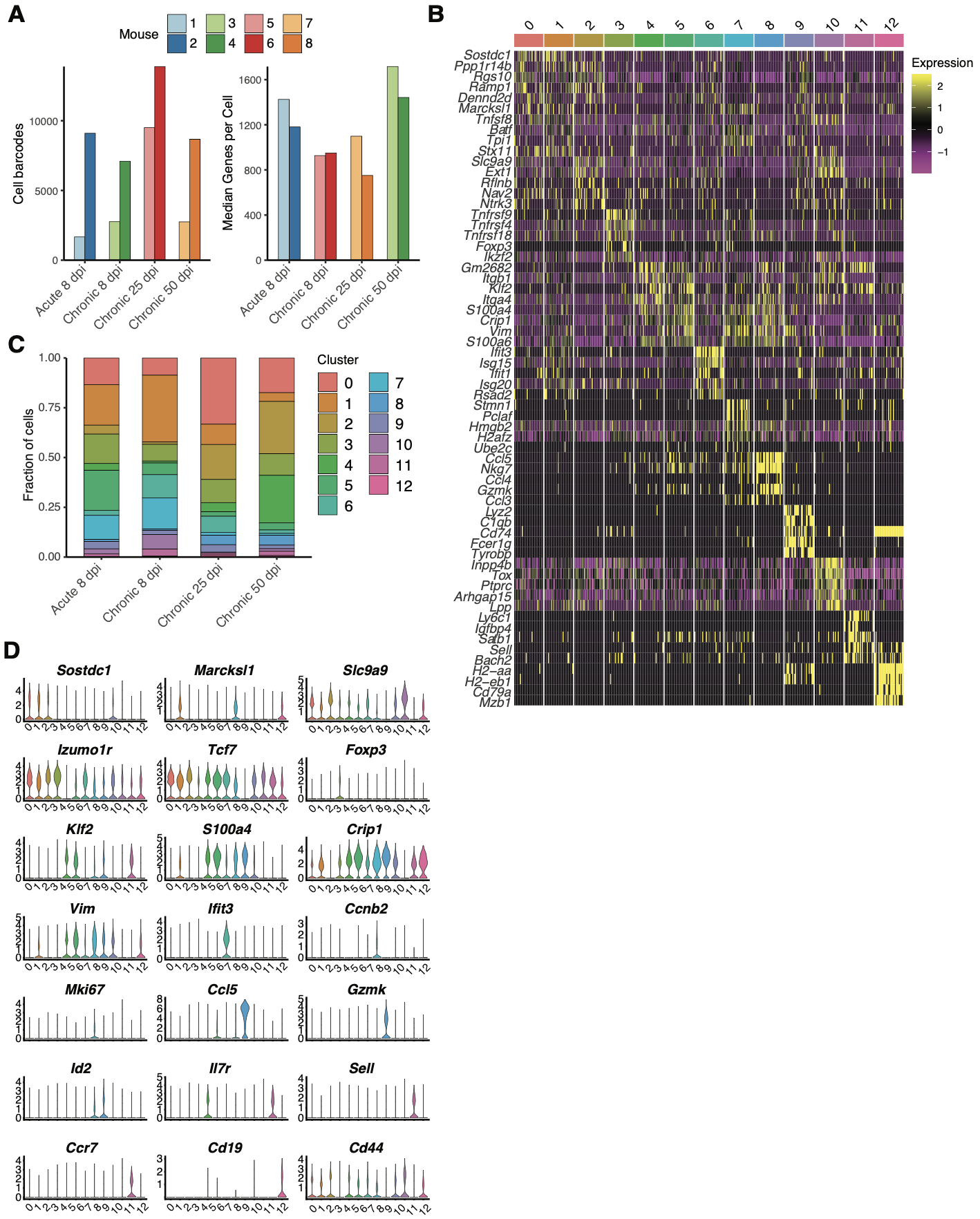
**

**Figure S1. Cluster defining gene signatures in Tfh cells after acute and chronic infection.** A. Number of cell barcodes with gene expression information for each mouse (left) and median number of genes per cell for each mouse (right). B. Fraction of cells belonging to each cluster for each experimental group. C. Top five significant genes defining each cluster ranked by average log fold change. D. Normalized expression for genes of particular interest in each cluster.

**
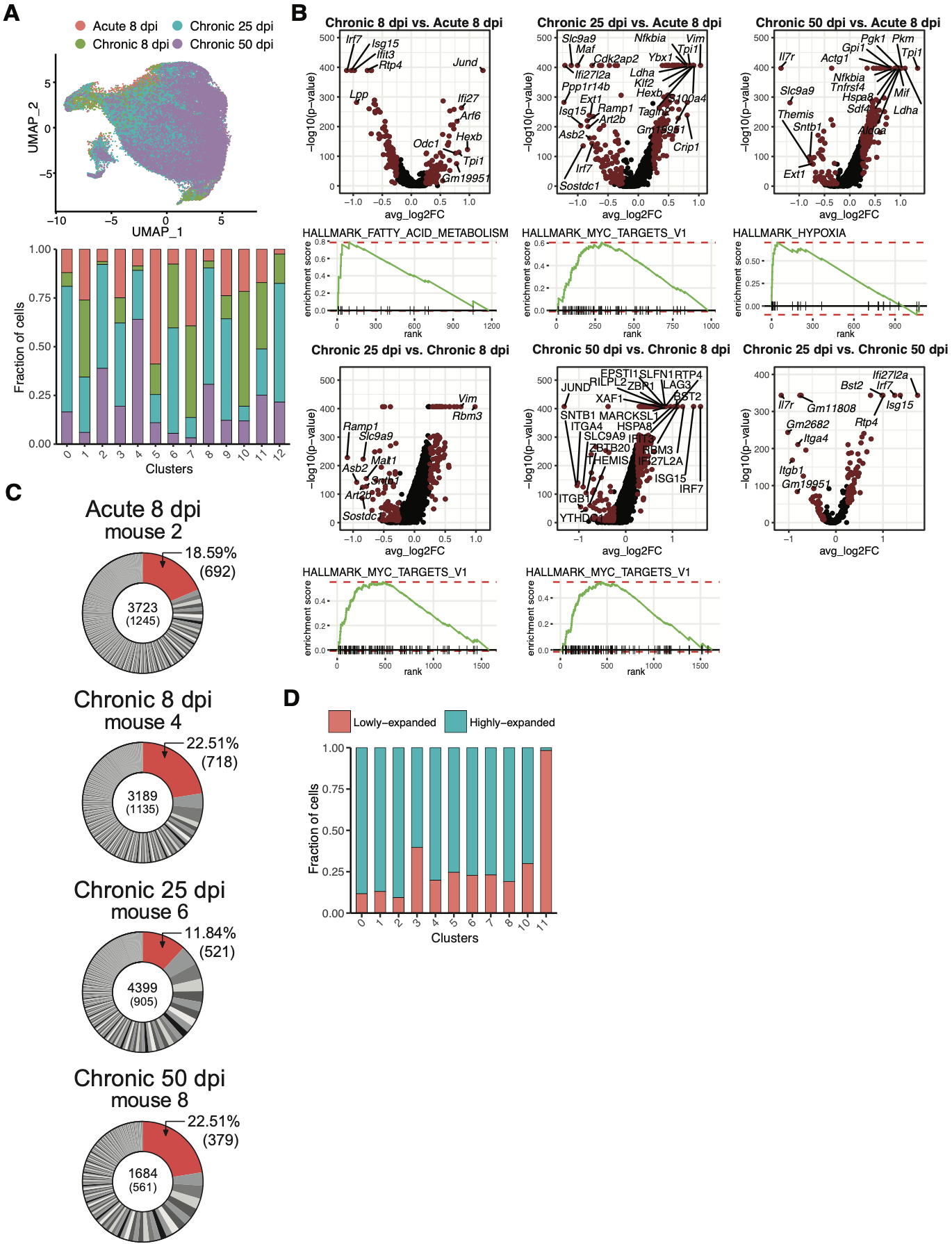
**

**Figure S2. Differential gene expression of Tfh cells during early and late LCMV infection.** A. Uniform manifold approximation projection (UMAP) displaying infection time point (top) and fraction of cells belonging to each infection time point in each transcriptional cluster. B. Differential gene expression and gene set enrichment (GSEA) analysis between acute LCMV 8 dpi and chronic LCMV 8 dpi (top left), acute LCMV 8 dpi and chronic LCMV 25 dpi (top middle), acute LCMV 8 dpi and chronic LCMV 50 dpi (top right), chronic LCMV 8 dpi and chronic LCMV 25 dpi (bottom left), chronic LCMV 8 dpi and chronic LCMV 50 dpi (bottom middle) and chronic LCMV 25 dpi and chronic LCMV 50 dpi (bottom right). Points in red indicate differentially expressed genes (adjusted p-value < 0.01 and average log_2_ fold change (FC) > 0.25). C. Residual donut plots showing distribution of clonal expansion in each infection time point. Each section corresponds to a unique clone (defined by CDR3α-CDR3β nt sequence) and the size corresponds to the fraction of cells relative to the total repertoire. Lowly-expanded clones (supported by only one unique cell) are colored in red. D. Fraction of lowly- and highly-expanded cells in each transcriptional cluster.


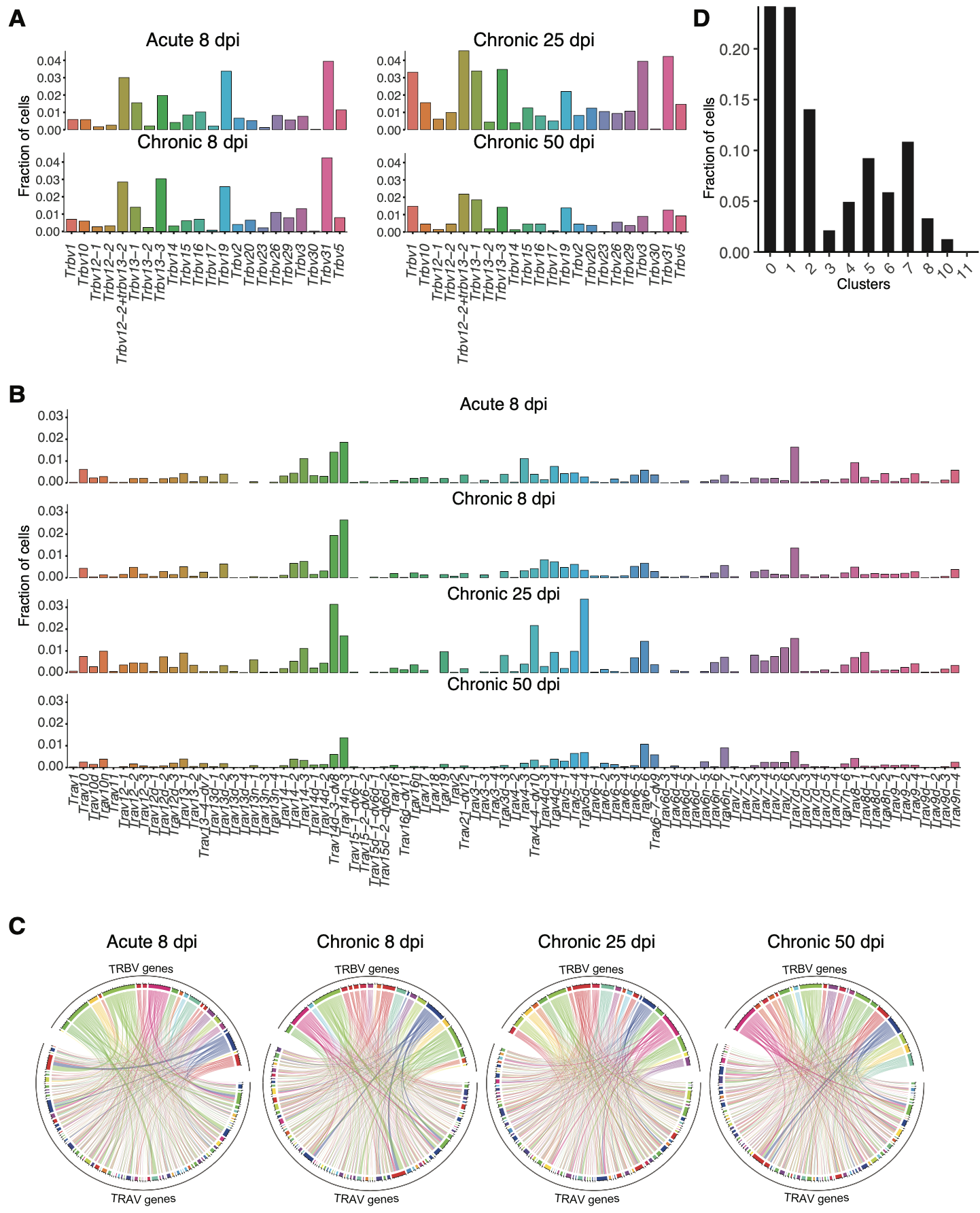


**Figure S3. Tfh cells during acute and chronic LCMV infection show similar abundance of TRAV and TRBV genes but no dominant pairing patterns.** A. TRBV germline gene usage within Tfh clones (CDR3α-CDR3β nt sequence). B. TRAV germline gene usage within Tfh clones (CDR3α-CDR3β nt sequence). C. Circos plots depicting the relationship between TRB and TRA V genes. Color corresponds to TRBV gene usage. Connections illustrate the number of cells using each particular combination. D. Fraction of shared clones within each transcriptional cluster.


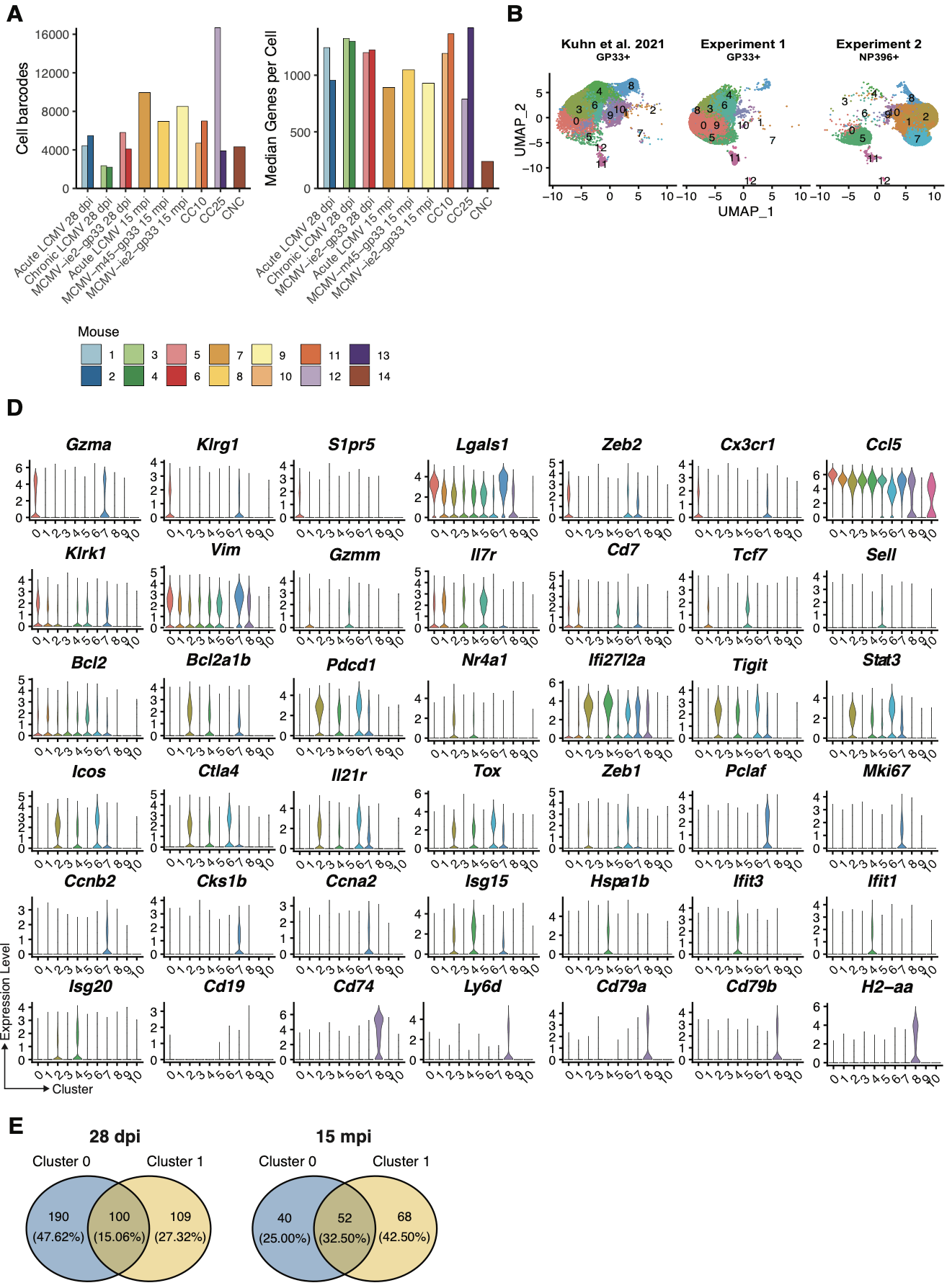


**Figure S4. Batch effect analysis and cluster defining gene signatures in virus-specific CD8+ T cells.** A**.** Number of cell barcodes with gene expression information for each mouse (left) and median number of genes per cell for each mouse (right). B. Uniform manifold approximation projection (UMAP) split by experiment. Data was integrated with the standard normalization method from the Seurat package. C. Normalized expression for genes of particular interest in each cluster.D. Venn diagram depicting number and percentage of differentially expressed genes shared between clusters of same-aged mice.


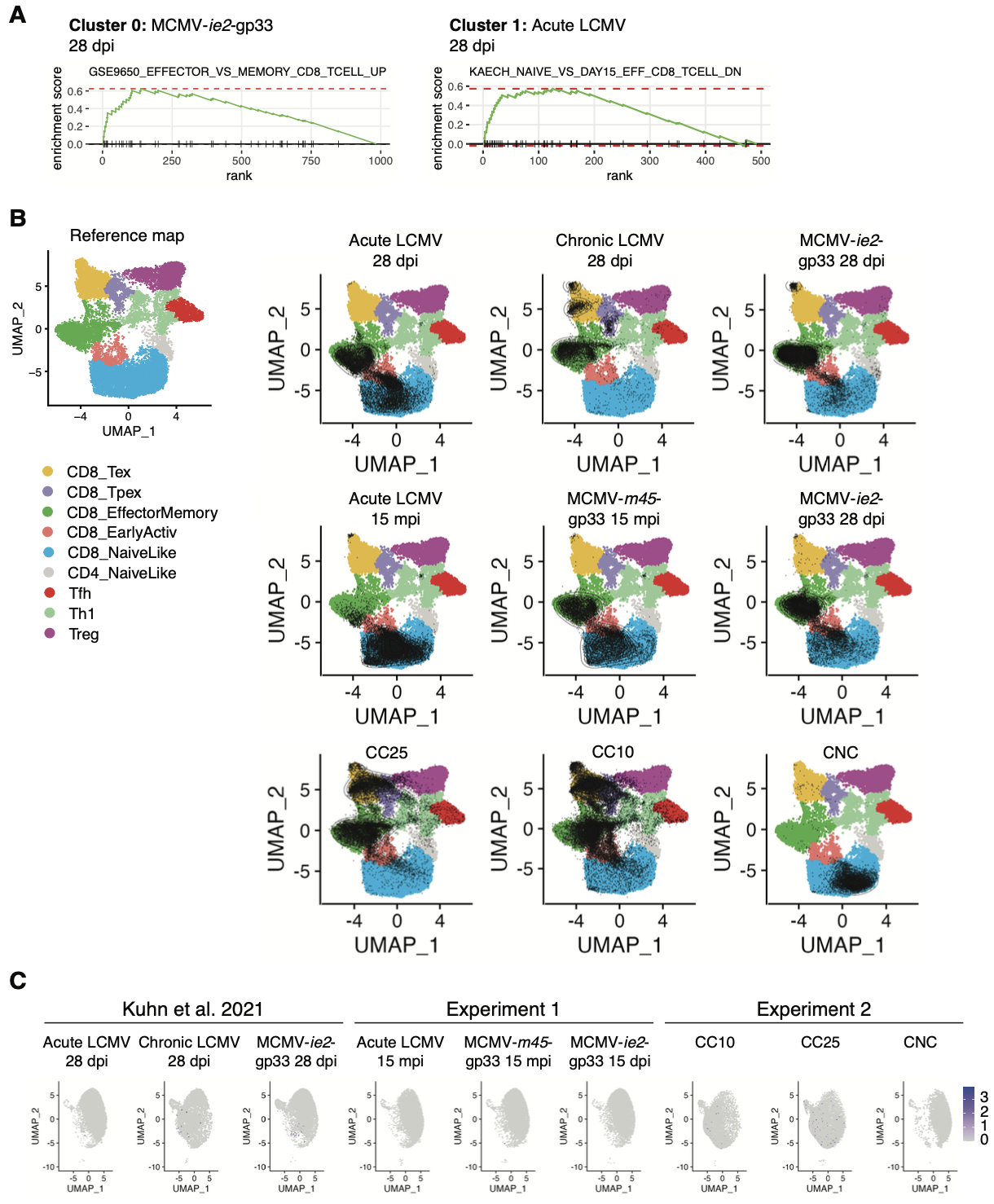


**Figure S5. Predicted Cell states and repertoire sequence motifs** **of virus-specific CD8+T cells and polyclonal Tfh cells.** A. Gene set enrichment (GSEA) analysis in cluster 0 MCMV-*ie2*-gp33 infection 28 dpi (left) and cluster 1 acute LCMV infection 28 (right). B. Single-cell projection of GP33- and NP396-specific CD8+ T cells on the default reference TIL atlas (54). C. Uniform manifold approximation projection (UMAP) split by infection type showing *Cd4* expression.


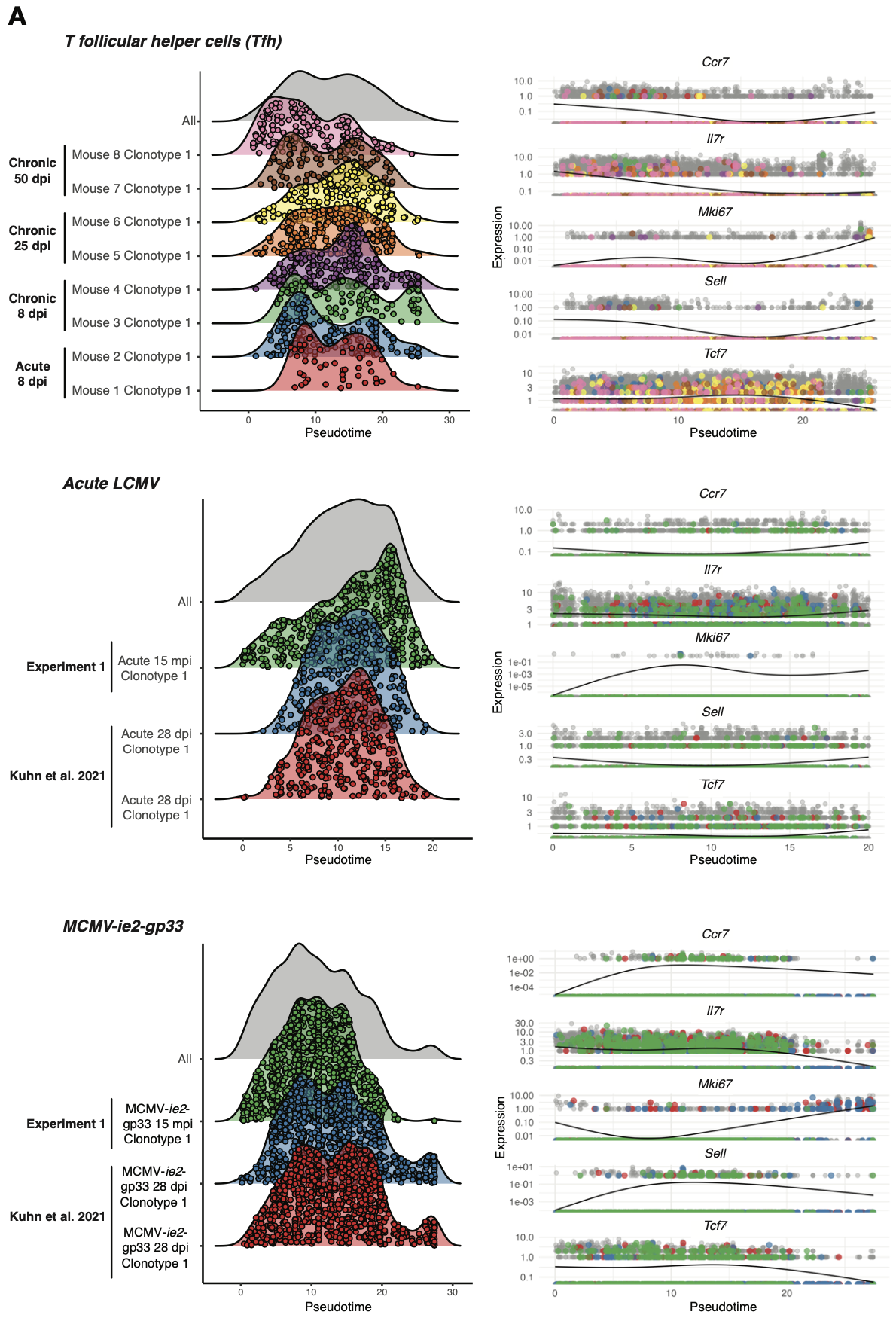


**Figure S6. Trajectory analysis of Tfh cells and virus-specific cells following acute LCMV and MCMV-ie2-gp33 infection.** A. Monocle-inferred distribution of pseudotime for the most expanded clones in each mouse (left) and Monocle-inferred pseudotime for select genes for the most expanded clones in each mouse (right). Each point represents a cell.


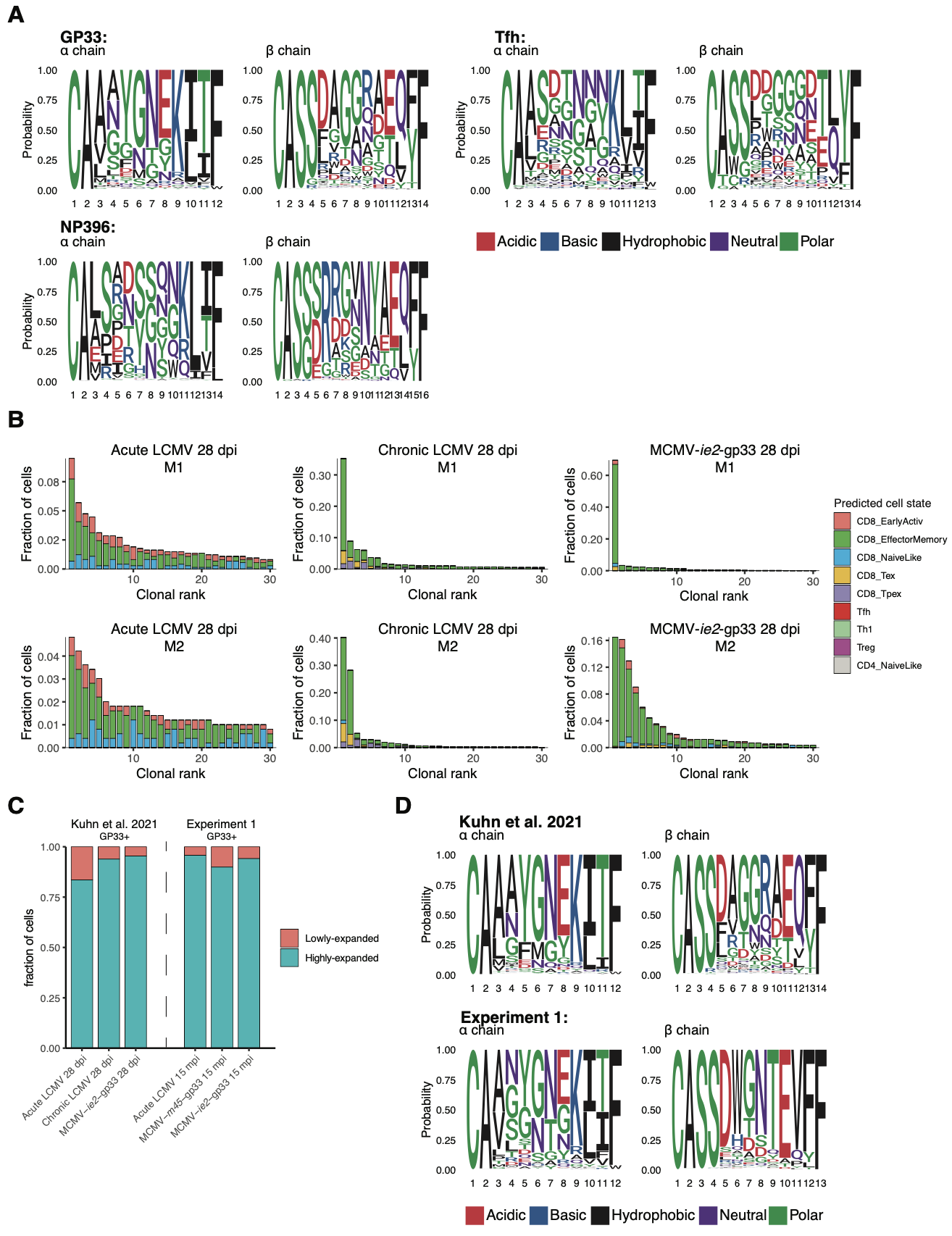


**Figure S7. Differences in clonal composition and expansion aged-matched virus-specific CD8 T cells following LCMV and MCMV infection.** A. Sequence logo plots of the alpha and beta chain of GP33- and NP396-specific CD8+ T cells and polyclonal Tfh cells. B. Top 30 most expanded clones of virus-specific CD8 T cells from Kuhn et al. separated by the predicted cell state using the nearest-neighbor classifier of the ProjecTILs algorithm (54). C. Fraction of lowly- and highly-expanded cells. C. Sequence logo plots of the alpha and beta chain of the aged-matched GP33-specific CD8+ T cells.


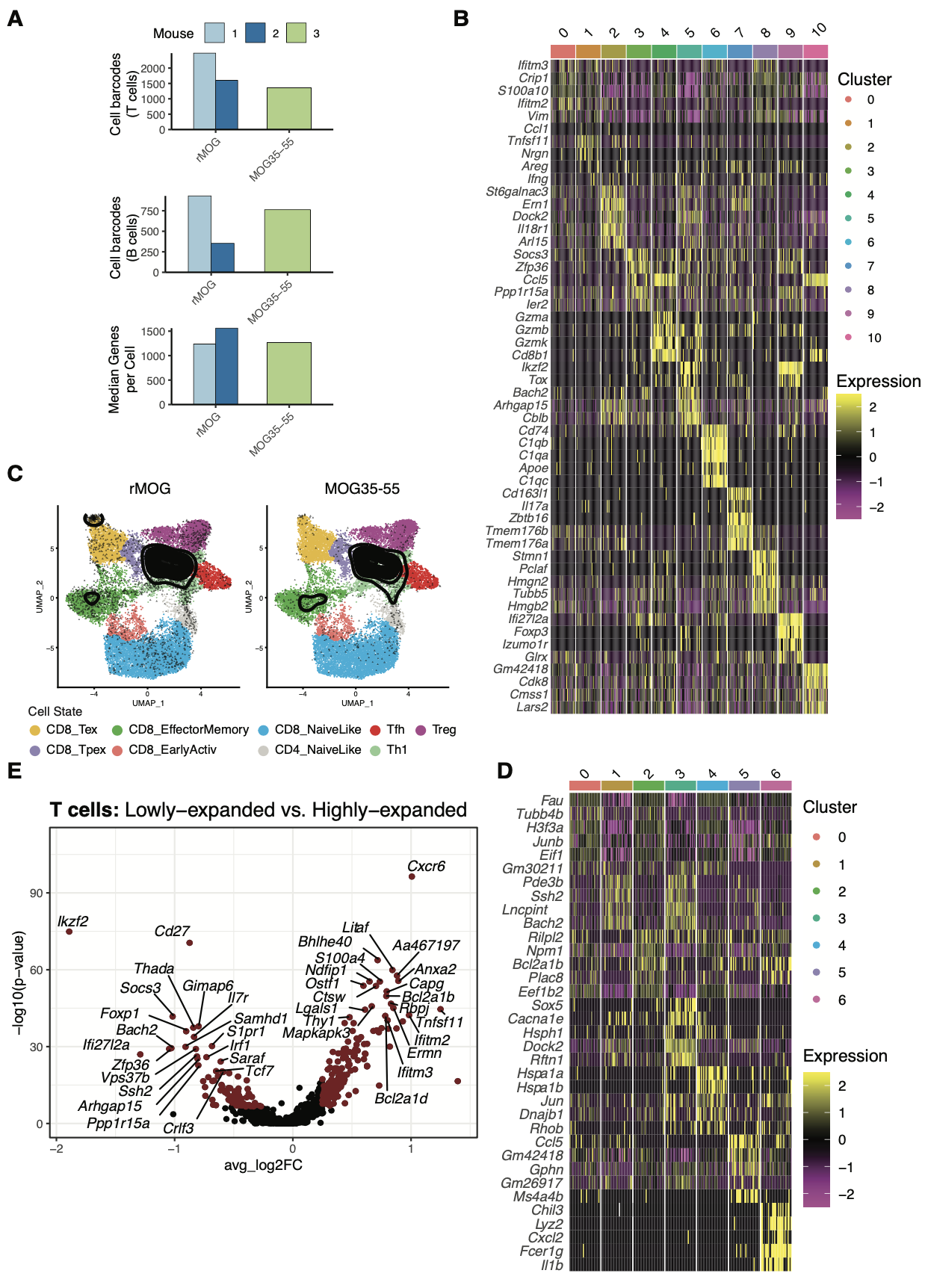


**Figure S8. Cluster defining gene signatures in B and T cells following experimental autoimmune encephalomyelitis (EAE) induction with either rMOG and MOG_33-55_.** A. Number of cell barcodes with gene expression information for each mouse and median number of genes per cell for each mouse. B. Single-cell projection of T cells following EAE induction with either rMOG and MOG_33-55_ on the default reference TIL atlas (54). C. Top five significant genes defining each cluster of T cells ranked by average log fold change. D. Differential gene expression between lowly-expanded (1 cell) and highly-expanded (more than one cell) CD4+ and CD8+ T cells E. Top five significant genes defining each cluster of B cells ranked by average log fold change.
